# Performance and mechanisms of enhanced hydrolysis acidification by adding different iron scraps: Microbial characteristics and fate of iron scraps

**DOI:** 10.3389/fmicb.2022.980396

**Published:** 2022-08-24

**Authors:** Yanqiong Wang, Hongwu Wang, Hui Jin, Hongbin Chen

**Affiliations:** ^1^State Key Laboratory of Pollution Control and Resource Reuse, National Engineering Research Center for Urban Pollution Control, College of Environmental Science and Engineering, Tongji University, Shanghai, China; ^2^Shanghai Institute of Pollution Control and Ecological Security, Shanghai, China

**Keywords:** hydrolysis-acidification, iron scraps, VFAs production, microbial community structure, redundancy analysis, molecular ecological network

## Abstract

HA, as one of low-carbon pre-treatment technology could be enhanced by packing of iron or iron oxide powder for enhancing the transformation of large molecular weight to generate volatile fatty acids (VFAs) for fuel production. However, the controversy of iron strengthening the HA and inherent drawbacks of iron oxide, such as poor mass transfer, and difficult recovery, limit this pretreatment technology. Clean and rusty iron scraps were packed into an HA system to address these issues while focusing on the system performance and the response of core bacterial and fungal microbiomes to iron scrap exposure. Results showed that clean and rusty iron scraps can significantly improve the HA performance while considering hydrolysis efficiency (HE), acidification efficiency (AE) and VFAs production, given that VFAs ratios (C_acetate_: C_propionate_: C_butyrate_) were changed from the 14:5:1 to 14:2:1 and 29:4:1, respectively, and the obtained VFAs ratios in iron scraps addition systems were more closely to the optimal VFAs ratio for lipids production. Redundant and molecular ecological network analyses indicated that iron scraps promote the system stability and acidogenesis capacity by boosting the complexity of microbes’ networks and enriching core functional microbes that show a positive response to HA performance, among which the relative abundance of related bacterial genera was promoted by 19.71 and 17.25% for R_Rusty_ and R_Clean_ systems. Moreover, except for the differences between the control and iron scraps addition systems, the findings confirmed that the R_Rusty_ system is slightly different from the R_Clean_ one, which was perhaps driven by the behavior of 6.20% of DIRB in R_Rusty_ system and only 1.16% of homoacetogens in R_Clean_ system when considering the microbial community and fate of iron scraps. Totally, the observed results highlight the application potential of the iron scrap-coupled HA process for the generation of VFAs and provide new insights into the response of different iron scraps in microbes communities.

## Introduction

The hydrolysis–acidification (HA) process is a widely used pre-treatment for wastewater containing compounds with large molecular weight (Lu et al., 2016; Xie et al., 2018). HA involves both hydrolysis and acidification procedures that transform complex macromolecules to small molecules. The HA process plays an important role in meeting the COD emission requirement given that the improvement of biodegradability is beneficial for the subsequent biological treatment system (Tian et al., 2019). Furthermore, the HA process is suitable for the concept of low carbon because it can generate VFAs. The VFAs can be used as raw chemical materials for lipid production, which can be derived into the promising fossil fuel alternative named biodiesel (Fei et al., 2011; Tharak and Venkata Mohan, 2021). It has been reported that microbial lipids could derived by heterotrophic microalgae such as *Chlorella* which could convert the carbon source such as glucose to acetyl-CoA and finally generate lipid (Fei et al., 2015). Compared with glucose, VFAs with suitable ratio were more efficient and economical since it could be generated from HA process of a variety of organic wastes (Schneider et al., 2013).

However, low HA efficiency due to the inhibited microbial activity limits the application of HA (Zhang et al., 2021). The addition of exogenous substances (including Fe° and iron oxides) can effectively improve the performance of the HA process. Fe° is a reliable, inexpensive materials that can promote the HA process by improving the activity of enzymes associated with the process when added to an anaerobic system (Meng et al., 2013; Hao et al., 2017). However, Zhao et al. (2018) reported that biological hydrolysis and the acid-producing process remain unaffected by the addition of Fe° to the waste-activated sludge digestion system. Therefore, investigating the effects on and mechanisms of Fe° addition in the HA performance is crucial.

Iron oxides exert positive effects on the HA process. A previous study demonstrated that Fe_2_O_3_ and Fe_3_O_4_ (Ye et al., 2018; Zhao et al., 2018) could remarkably promotes the HA process. The potential mechanisms are presented as follows: iron oxides can enrich dissimilatory iron-reducing bacteria (DIRB) to couple the oxidation of complex organics and reduce insoluble iron oxides via the dissimilatory iron reduction (Light et al., 2018). However, studies on the optimization of the VFA ratio, which is important to fuel production, are limited. Compared with iron oxides, rusty iron scraps covering the iron oxide layer on the surface were selected due to their low cost and excellent mass transfer. In addition, iron shavings demonstrate advantages in recycling and reusing because of the low utilization of iron oxides (Wang M. et al., 2019). However, information on the coupling of rusty iron shavings in the HA process for macromolecule bioremediation is still limited.

Additionally, HA sludge is a highly complex ecosystem of bacteria and fungi, which coexist within complicated networks with a multitude of interactions. Succession, identification of interaction between microorganisms, and keystone species of microorganisms are important in obtaining new insights into the HA process. However, bacterial, and fungal communities under iron shaving simulation still remain unclear. Researchers have recently applied redundancy analysis (RDA) to test the correlation between environmental factors and microbes statistically and provide evidence for the correlation between microbial community succession and system performance (Chen et al., 2021). Moreover, molecular ecological networks (MENs) can describe potential interactions of complex microbial communities and identify the keystone species in various environments (Wang X. et al., 2019; Chen et al., 2021).

Thus, artificial wastewater containing dextran (Mw = 200 kDa) was selected to simulate the wastewater containing macromolecular organic matters, such as molasses fermentation wastewater. Clean and rusty iron craps were dosed into the HA process in this study to explore the effects of iron craps on HA process for the pre-treatment of wastewater containing macromolecular organics from the aspects of HA performance. Sludge characteristics and succession of bacterial and fungal communities were explored from aspects of community constructure, correlation between environmental factors and microbes, interactions networks of different functional microorganisms, and fates of different iron scraps to explore the effect mechanisms.

## Materials and methods

### Preparation of iron scraps

Two kinds of iron scraps were used in this study: clean and rusty iron scraps. Iron scraps (38CrMoAl) with a spirally curved shape and a length of about 30 cm were collected from a mechanical factory. The iron scraps are cut into 3 cm-long pieces to increase the specific surface area and improve mass transfer rate. Li et al. (2019) soaked the collected iron scraps in 1 mol/L NaOH solution for 24 h to remove oil stains, washed them with deionized water to use, immersed them in 0.1 mol/L HCl solution for 0.5–1 h to remove the surface rusty layer, and then washed them again with deionized water to use immediately. Meanwhile, rusty iron scraps were placed in a humid environment until the surface layer is covered in rust.

### Seed sludge and artificial wastewater

The original sludge was obtained from Quyang Wastewater Treatment Plant (Shanghai, China). Sludge (250 mL) with 4 g/L of MLSS was inoculated into three reactors after 2 weeks of acclimation. The main parameters of the artificial wastewater used in the system were as follows: a mixture of glucose and dextran corresponding to 1,000 mg/L of chemical oxygen demand (COD) was used as the organic carbon source, 127 mg/L of NH_4_Cl and 29.2 mg/L of K_2_HPO_4_ were added to obtain a COD/N/P ratio (mass ratio) of 150:5:1, and 500 mg/L of NaHCO_3_ was used as the buffer to maintain a pH level close to 8.0. The trace element composition is consistent with Supplementary Table 1.

### Setup and operation of reactors

Three polymethyl methacrylate cylindrical sequential batch reactors (SBRs) with a working volume of nearly 500 mL (φ100 mm × 150 mm) were used. Similar to the method of Zhao et al. (2018), 10 g/L of clean iron scraps prepared in section “Preparation of iron scraps” were placed at the bottom of the reactor labeled R_Clean_ to avoid exposure of iron scraps to air and prevent oxidation. Rusty iron scraps (10 g/L) were placed in the middle of the reactor labeled R_Rusty_ to allow exposure of iron scraps to air during water replacement and maintain the rusty layer continuously.

All reactors were operated at room temperature in the sequencing batch mode of a 12-h cycle consisting of filling (0.1 h), stirring (10 h), settling (0.5 h), decanting (0.1 h), and idling (1.3 h). Influent was added from the top of reactors, while effluent was controlled using a valve at the side of the reactor for analysis.

### Analytical methods and data analysis

#### Analytical methods

Water quality parameters (COD and TP) and sludge properties [mixed liquid (MLSSs) and mixed liquid volatile (MLVSSs) suspended solids] were measured using standard methods (APHA, 1998). The pH level was monitored using a pH meter (PHSJ-3F). Tian et al. (2021) determined the concentrations of Fe^2+^ using phenanthroline spectrophotometry. Molecular weights and their distributions were examined via gel chromatography (Aglient 1260). Volatile fatty acids (VFAs) were assessed through gas chromatography (GC, Aglient GC-6890N/FID). Dehydrogenase activity (DHA) was explored using TTC spectrophotometry (TU-1810) according to Wang et al. (2021).

EPS was extracted using the cation exchange resin, and the content of polysaccharide (PS) and protein (PN) was tested through Lowry and phenol–sulfuric acid methods. The 3D-EEM spectra of EPS samples were measured with a HORIBA fluorescence spectrometer.

The morphology and surface elements of iron scraps and the sludge were examined using scanning electron microscopy (SEM) and energy dispersive spectroscopy (EDS). The microbial community was tested with the 16S rRNA gene high-throughput sequencing Illumina MiSeq platform. RDA was conducted via Caonon 4.5.

#### Data analysis

Hydrolysis efficiency (HE) can be expressed as follows:


(1) HE(%)=(1-PercentageofMw>100kDa/(50%))×100%

where 50% is the percentage of Mw > 100 kDa in the influent.

Acidification efficiency (AE) can be expressed as follows:


(2) $$\text{AE}\left(\%\right)=COD_{V\,F\,A\,s}/COD_{I\,n\,f\,l\,u\,e\,n\,t}\times 100\%$$

where COD_Influent_ is the concentration of influent COD (mg/L) and COD_VFAs_ is the concentration of effluent VFAs (mg/L COD). COD equivalents of each VFA are acetate, 1.07; propionate, 1.51; and butyric acid, 1.82 (Wang Y. et al., 2022).

According to Chen et al. (2021), RDA was applied to reveal the correlations between the environmental factors and bacterial and fungal community by using CANOCO 4.5. Co-occurrence networks were built using molecular ecological network analysis (MENA) to understand the interaction among microorganisms.

## Results and discussion

### Influence of different types of iron scraps on hydrolysis–acidification performance

#### Effects of different iron scrap addition on the hydrolysis process

**FIGURE 1:**
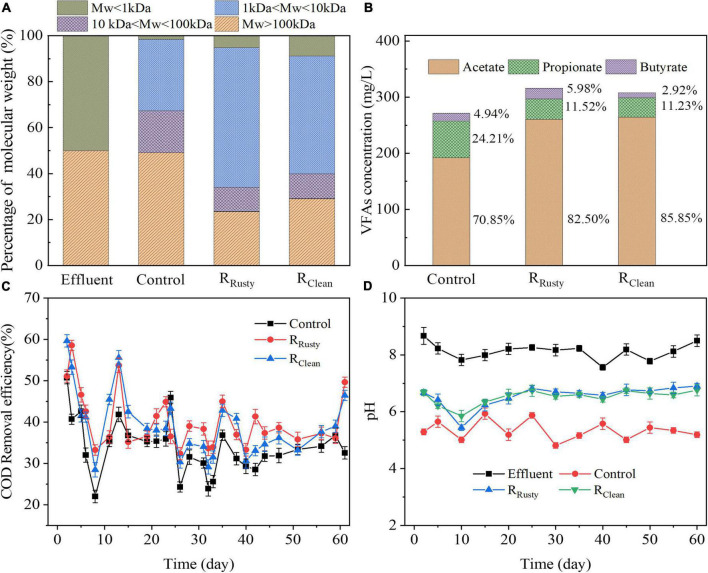
HA performance: **(A)** distribution of molecular weight; **(B)** VFAs concentration; and **(C)** COD removal, and **(D)** pH values in control, R_Rusty_, and R_Clean_ systems.

The hydrolysis process plays an important role in decomposing complex macromolecular organic substrate (e.g., PN and PSs) into soluble monomer or dimer (Shi et al., 2022) and is regarded as a rate-limiting step in anaerobic digestion due to the difficulty of the process (Liu et al., 2012). The distribution of molecular weight in the influent and effluent was analyzed in this study to evaluate the hydrolysis process (Figure 1A). The percentage of M_W_ > 100 kDa of the control group was about 49.08%, which is significantly higher than that of R_Rusty_ (23.39%) and R_Clean_ (29.06%) systems. Hence, the HE of R_Rusty_ (53.22%) and R_Clean_ systems (41.88%) was significantly higher than that of the control group (2.00%). These results indicated that the iron scrap addition enhances the hydrolysis of macromolecule organics by changing them into small-molecule organics and rusty scraps are more effective than clean iron scraps.

#### Effects of the addition of iron scrap on the acidification process

Saccharides with small molecular weight were generated and then converted into VFAs in the hydrolysis of PSs. VFAs are a critical factor that were detected in systems (Figure 1B). Average VFAs concentrations in control, R_Rusty_, and R_Clean_ system effluents during the operation period were 271.21, 308.87, and 307.70 mg/L, respectively. The dominant VFAs were acetate, propionate, and butyrate in this work which is consistent with the report of Liu et al. (2012). AE of the control group was the lowest at 32.9%, followed by R_Clean_ (35.1%) and R_Rusty_ (36.8%) systems. This finding indicated that iron scraps are beneficial for the acidification process, especially for rusty iron scraps. Notably, VFAs components were slightly different in systems. Average ratios of propionate in VFAs of control, R_Rusty_, and R_Clean_ systems during the HA process were 24.21, 11.52, and 11.23%, respectively. The acetate ratio of these three systems increased from 70.85 to 82.50% and 85.85%. The VFAs ratio for control was approximately 14:5:1 while R_Rusty_, and R_Clean_ systems were 14:2:1 and 29:4:1, respectively, which were closer to the optimal VFAs ratio (8:1:1) of lipid generation (Fei et al., 2015) given that the increase in the acetate ratio was beneficial to fuel production (Liu et al., 2016). These results suggested that the addition of iron scraps can enhance the acetate generation and optimize the VFAs ratio for fuel production. Previous studies reported that iron oxides can enhance the acetate production in the acidification process by promoting the dissimilatory iron reduction (Zhao et al., 2018; Xu et al., 2020). Hence, the enhancement of acetate production in the R_Rusty_ system in this work was likely due to the promotion of the dissimilatory iron reduction process by rusty iron scraps. Zhao et al. (2018) demonstrated that the acidification process in the R_Clean_ system remains unaffected by Fe°. By contrast, the acidification process was also promoted in this work. Fe° dosing is beneficial for the generation of butyrate and acetate because of the increasingly reductive environment (Feng et al., 2014; Dai et al., 2022). However, the production of butyrate did not improve in the R_Clean_ system in this work. Thus, compared with the reductive environment, the homoacetogenesis process might be the main reason. It was suggested that corrosion of clean iron scraps provides hydrogen (Eq. 3) to homoacetogens, which can use H_2_ to produce acetate through the homoacetogenesis process during acidification (Eq. 4) (Dong et al., 2022). Moreover, the consumption of H_2_ promoted the conversion of propionate to acetate by reducing the H_2_ content (Meng et al., 2013). Therefore, the generation of propionate reduced and the generation of acetate increased in the system with clean iron scrap addition in this study.


(3) $$\text{2}H_{\text{2}}O+Fe\to H_{\text{2}}+Fe^{\text{2}}{}^{+}+\text{2}OH^{–}$$

(4) $$\text{2}CO_{\text{2}}+\text{4}H_{\text{2}}\to CH_{\text{3}}COOH+\text{2}H_{\text{2}}O$$

#### Organic removal performance

COD removal efficiency is a main factor that can effectively evaluate the biological treatment process (Wang et al., 2008). The average COD removal efficiency was 32.62, 40.20, and 39.26% for the control, R_Rusty_, and R_Clean_ systems, respectively. Compared with that of the control group, the COD removal efficiency of R_Rusty_ and R_Clean_ systems increased by 7.58 and 6.64%, respectively. This finding indicated that the addition of iron scraps improves the removal of organic pollutants in the HA system (Figure 1C). Wang et al. (2008), Chen et al. (2012), and Wu et al. (2015) reported that the COD removal efficiency of the HA process is approximately 10.9, 26.9, and 30% when treating petrochemical, jean-wash, and sweet potato starch wastewaters, respectively. These results suggested that the addition of iron scraps promotes the COD removal and rusty iron scraps are beneficial for the HA performance.

#### Self-buffering capability of systems

Stable pH is an important factor in controlling the production of VFAs during fermentation (Lee et al., 2014). The stable neutral condition contributes to the high hydrolysis–acidification efficiency during the anaerobic digestion process of swine manure (Lin et al., 2013) and kitchen waste (Wang et al., 2016). The influent pH stabilized between 7.5 and 8.5 but the effluent pH of the three reactors differed throughout the operation period (Figure 1D). The effluent pH in the control group fluctuated between 4.81 and 6.46, with an average of 5.42, while that in the R_Rusty_ system varied between 6.12 and 6.90, with an average of 6.51, and that in the R_Clean_ system changed between 6.23 and 6.76, with an average of 6.53. This finding indicated that systems with additional iron scraps exhibit better pH self-buffering capability than systems without iron which was similar to the conclusion reported by Zhang Y. et al. (2020).

Regarding as R_Rusty_, Dong et al. (2016) and Zhang Y. et al. (2020) reported that hematite and ferrihydrite reduction can act as a pH buffer against acidification in R_Rusty_ systems due to the VFA accumulation from the consumption of protons (Eq. 5). Thus, the stability of pH and the enhanced self-buffering capability in the R_Rusty_ system was due to the iron oxides reduction.


(5) 8Fe(III)+24H++8e→-8Fe+2+24HO2

In terms of R_Clean_, iron scraps can be approximated as iron carbon micro-electrolysis material due to the existence of carbon in it. Fe° and carbon served as the sacrificial anode and cathode, respectively, and many microcurrent batteries spontaneously form with a series of chemical reactions (Eqs. 6, 7) (Chen et al., 2011; Hwang et al., 2019; Li et al., 2021). Thus, the balance between the continuous consumption of protons and acidification maintained the stability of pH in the R_Clean_ system.


(6) Anode:Fe-2e=-Fe+2

(7) Cathode:2H++2e→-H2

### Strengthening effects of different iron scraps on hydrolysis–acidification sludge

#### Characterization of sludge surface

Sludge samples from control and iron scrap addition groups were characterized via SEM-EDS to examine surface changes and determine the elemental composition of sludge. Supplementary Figure 1 shows the SEM images and EDS spectra of sludge samples from control, R_Rusty_, and R_Clean_ systems. A mixture of cells with bacilli and coccus-shaped morphology clearly coexisted. Moreover, EPS were observed and tiny particles deposited on the surface of cells, particularly in SEM images of R_Rusty_ (Supplementary Figure 1C) and R_Rusty_ (Supplementary Figure 1E) systems. Notably, EPS can serve as a potential flocculating agent for heavy metal precipitation, including Fe (Siddharth et al., 2021). The EDS analysis showed that the spectra in Supplementary Figures 2D,F reveal peaks for C, O, Fe, and P in sludge samples from R_Rusty_ and R_Clean_ systems. C and O are major components of cells (Zacarías-Estrada et al., 2020). Considering the peaks for Fe and P elements, the TP removal efficiency, and the solution TFe concentration (Supplementary Figure 2) were detected, the TP was removed simultaneously in HA systems by the formation of precipitates (P–Fe).

#### Extracellular polymeric substances

On the basis of section “Characterization of sludge surface,” the measured EPS content in all bioreactors at the end of experiments is listed in Supplementary Table 2. EPS concentrations were 49.24 and 49.31 mg/gVSS in R_Rusty_ and R_Clean_ systems, which was higher than those in the control system by 6.3 and 6.5%, respectively. Erdim et al. (2019) and Zhang D. et al. (2020) reported that microorganisms increase the production of EPS in response to nanoscale zero-valent iron. These results suggested that the addition of iron can increase the production of EPS. Notably, EPS secreted by microorganisms plays an important role in the structural stability of the sludge (Liang et al., 2021) and the PN content in R_Rusy_ (17.39 mg/gVSS) and R_Clean_ (17.38 mg/gVSS) systems were higher than that in the control system14.09 mg/gVSS. A previous study showed that the increase of PN content can enhance the flocculation ability of EPS and subsequently improve the stability of the system (Siddharth et al., 2021). Therefore, improved stability in R_Rusty_ and R_Clean_ systems described in section “Organic removal performance and Self-buffering capability of systems” may be due to the increased production of EPS and PN.

#### Enzyme activity

**FIGURE 2:**
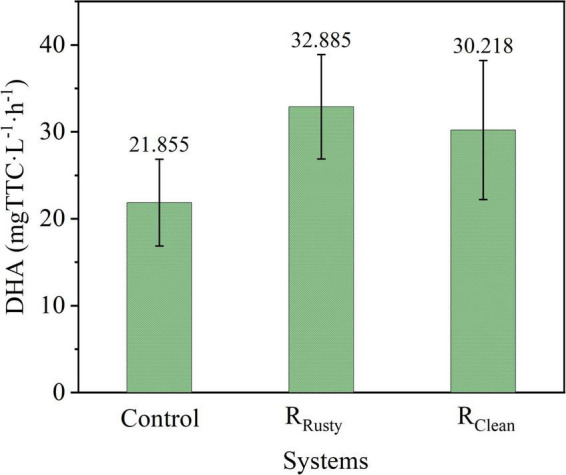
DHA in control, R_Rusty_, and R_Clean_ systems.

Microorganism activity is a key factor during biological treatments (Boyd and Shelton, 1984; Hongwei et al., 2002), and dehydrogenase is necessary for microbe survival (Goel et al., 1998; Zhang et al., 2018). Average DHAs of three systems within 60 days are illustrated in Figure 2. DHA values were 21.855, 32.885, and 30.218 mg TTC (L^–1^⋅h^–1^). Hence, the respective DHA values of R_Rusty_ and R_Clean_ systems were 50.47 and 38.26% higher than those in the control group. Tian et al. (2021) and Wang et al. (2021) demonstrated that iron foam and Fe/C can facilitate dehydrogenase secretion and improve the microbial activity, respectively. These results suggested that iron dosing enhances DHA and thus improves the microorganism activity. Notably, Fe^2+^ can penetrate cells and promote the synthesis of key enzymes (Zhu et al., 2014; Ou et al., 2016). Therefore, the enhancement of DHA in this work was mainly due to the released Fe^2+^.

### Microbial community analysis

#### Bacterial community

**FIGURE 3:**
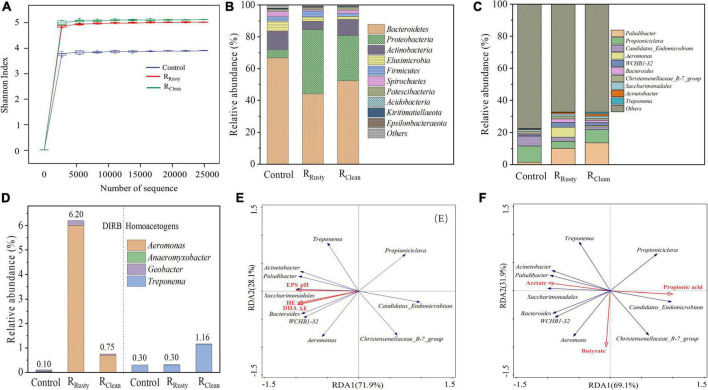
Bacterial communities. **(A)** Shannon curves; **(B,C)** microbial community structure at the phylum and genus levels; **(D)** relative abundance of dissimilatory iron-reducing bacteria (left) and homoacetogens (right) in control, R_Rusty_, and R_Clean_ systems; and **(E,F)** redundancy analysis revealed the correlation between the microbial community and environmental factors for sludge samples.

Shannon index was calculated to reveal community diversities, including evenness and richness. The sample from HA systems with additional iron scraps presented higher bacterial community diversity than that from the control system (Figure 3A). The improvement of ecological stability from high biodiversity (Chen et al., 2021) suggested that the addition of iron scraps is beneficial for the HA system.

Microbial communities of acclimated sludge obtained from control, R_Rusty_, and R_Clean_ systems were analyzed on day 60. The microbial community structure at the phylum level is shown in Figure 3B. The main phyla in these sludge samples Proteobacteria, Firmicutes, Bacteroidetes, and Actinobacteria accounted for 86.48% (control system), 93.62% (R_Rusty_ system), and 93.07% (R_Clean_ system) of the microbial population. The correlation between these phyla and the HA process (Xie et al., 2018; Yang et al., 2019) indicated that the improvement of the HA process from the addition of iron scraps is likely due to the enrichment of phyla.

Further genus-level analysis revealed that DIRB in this study covers different taxa of Firmicutes, Proteobacteria, and Actinobacteria (Esther et al., 2015). Figure 3D illustrates that the total abundance of DIRB in the control system is 0.1% while the addition of iron scraps in the HA system increases the abundance of DIRB to 6.20 and 0.75% in R_Rusty_ and R_Clean_ systems, respectively. A previous study proved that *Geobacter* and *Shewanella* are two typical DIRBs that can oxidize complex organic matter and reduce insoluble iron oxides to generate solution Fe^2+^ via extracellular electron transfer (EET) (Esther et al., 2015). Huang et al. (2019) has proved the capability of EET in *Aeromonas*, which is the dominant DIRB in R_Rusty_ and R_Clean_ systems, with a relative abundance of 6.0 and 0.70%, respectively, thereby indicating that types of iron scraps significantly influence the relative abundance of DIRB. Zhao et al. (2018) reported that iron oxides can promote the growth of DIRB during the anerobic digestion of waste active sludge. Therefore, the continuously generated layer of rusty iron composed of iron oxides can also play an important role in the enrichment of DIRB in the R_Rusty_ system. In terms of R_Clean_ system, Tian et al. (2021) showed that sludge can facilitate iron oxidation to generate iron oxides, thereby indicating that trace contents of iron oxides produced by microbial corrosion may be the main reason for the DIRB abundance of 0.75% but is negligible compared with the R_Rusty_ system.

The relative abundance of homoacetogens was also observed in this work (Figure 3D). *Treponema* was the dominant homoacetogen, with a relative abundance of 0.3, 0.3, and 1.16% in control, R_Rusty_, and R_Clean_ systems, respectively. *Treponema* used CO_2_/H_2_ to produce acetate on the basis of reaction (6) (Yang et al., 2021). Thus, high acetate production in the R_Clean_ system may be due to the relative abundance of *Treponema*.

The correlation between microbial communities and the system metabolite was analyzed through RDA. Five parameters of the HA system, namely, pH, HE, AE, EPS, and DHA, were subjected to RDA together with the top 10 genera of bacteria. As shown in Figure 3E, *Paludibacter, Aeromonas, WCHB1-32, Bacteroides, Saccharimonadales*, *Acinetobacter*, and *Treponema* all showed a positive correlation with HE, AE, and EPS production as well as DHA given that the sharp angle between environmental factors and the above genus, which accounts for 4.42, 24.13, and 21.66% of control, R_Rusty_, and R_Clean_ systems. Moreover, *Bacteroides* was closely related to HE and AE because of its excellent hydrolysis and acidification capacity (Zhou et al., 2016). The high relative abundance of *Bacteroides* in R_Rusty_ and R_Clean_ systems indicated its benefits for the HA process (Figure 3C). The composition of VFAs, including acetate, propionate, and butyrate, in the HA system were subjected to RDA together with the top 10 genera of bacteria to obtain new insights into the correlation between VFA generation and microbes. Figure 3F shows that the positive response of *Treponema* to acetate generation is due to the homoacetogenesis process. *Paludibacter*, which can also produce acetate (El-Bery et al., 2013), presents a positive correlation to acetate. The relative abundance of *Paludibacter* in HA systems with iron scraps was higher than that in the control system by 8.7 and 12.1%, providing strong evidence for the high production of acetate in HA systems with additional iron scraps. *Propioniciclava* showed a positive response to the propionate because it can ferment carbohydrates to produce propionic acid (Sugawara et al., 2011), and the decrease of relative abundance of that can explain the low yield of propionic acid in HA systems with additional iron scraps.

#### Fungal community

**FIGURE 4:**
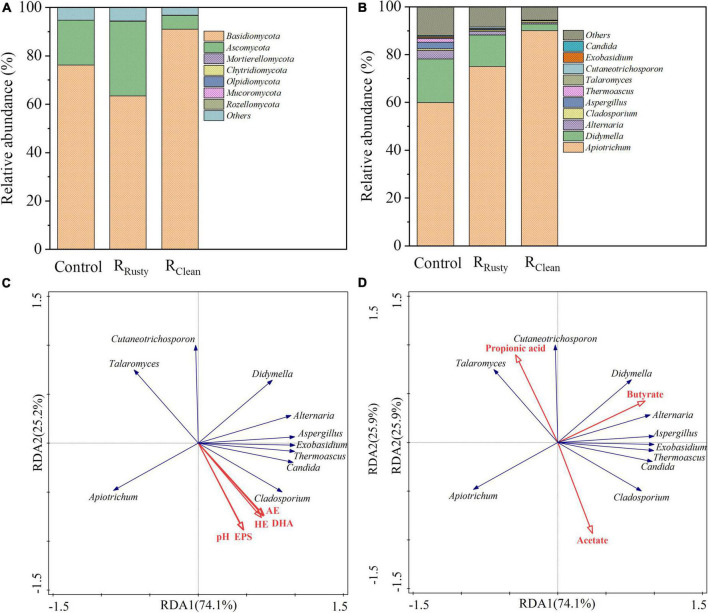
Fungal communities. **(A,B)** Microbial community structure at the phylum and genus levels and **(C,D)** correlation between the microbial community and environmental factors for sludge samples based on redundancy analysis.

Basidiomycota and Ascomycota were determined in sludge samples from HA systems (Figure 4A), their total abundance was over 90%, and the addition of iron scraps slightly promoted their enrichment. Chen et al. (2021) reported that the phyla Basidiomycota and Ascomycota play key roles in the degradation of complex organic pollutants, such as polymeric carbohydrate substance. The abundance of the top 10 fungal genera is presented in Figure 4B. The main fungal genus in HA systems was *Apiotrichum*, which is related to the biotransformation of complex organic substances. Its related abundance was also enriched by 15.0 and 30.0% with the addition of rusty and clean iron scraps, respectively. Five parameters of the HA system, including pH, HE, acidification efficiency AE, EPS, DHA, and VFAs, were also subjected to RDA together with the top 10 genera of fungus. Figures 4C,D illustrate the positive response of *Apiotrichum* to metabolic and environmental factors related to HA performance. Meanwhile, the relative abundance in three systems can provide evidence for the enhancement of the HA performance with the addition of iron scraps. *Cladosporium* showed a strong response to the HA physicochemical property and metabolite likely because *Cladosporium* is an important portion of the overall fungal community that degrades complex organic compounds during sludge anerobic digestion (Sun et al., 2015). However, this phenomenon was not discussed in detail given the similar relative abundance values of the three systems.

#### Iron scrap addition altered microbial co-occurrence networks

**FIGURE 5:**
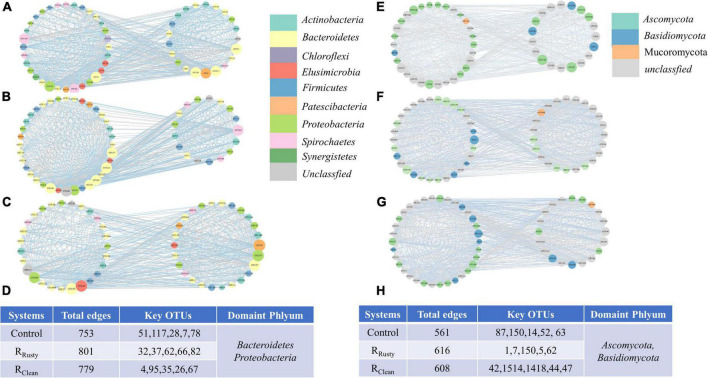
**(A–D)** Bacterial and **(E–H)** fungal ecological networks of sludge in **(A,E)** control, **(B,F)** R_Rusty_, and **(C,H)** R_Clean_ systems. Each node represents an OTU, and the size represents the number of lines. Different colors represent various species classifications (phylum level).

Iron scraps remarkably increased the network size in terms of the total edges in networks of R_Rusty_ and R_Clean_ systems with high amounts of edges (Figures 5D,H). The top five nodes with high connectivity in each system were explored. The simplest network was the control network with 238 and 172 links to first neighbors (Figures 5A,E), followed by R_Clean_ (243 and 195 links) (Figures 5B,F) and R_Rusty_ (246 and 193 links) (Figures 5C,G) systems, respectively. This finding indicated that iron scraps increase the complexity of the microbial network. Increasingly complex network structures of sludge samples can be a potential factor for the enhanced HA performance among the three systems because the biodiversity of interaction types can effectively enhance the system stability by increasing the resistance ability to environmental factors (Wang J. et al., 2022). The top five highly linked nodes in each network were only shared sometimes among the three networks, indicating that iron scraps and its types affect the overall architecture of the network. However, the top five highly linked nodes were all classified as phyla *Bacteroidetes, Proteobacteria*, *Basidiomycota*, and *Ascomycota*, thereby indicating the importance of these phyla during the HA process.

**FIGURE 6:**
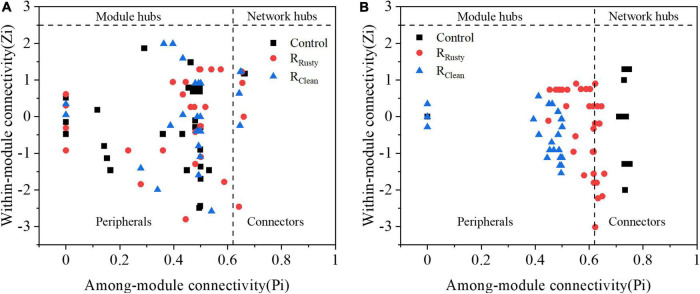
Node classification to identify putative keystone species within sludge system networks. **(A)** Bacterial and **(B)** fungi. [All nodes were divided into four categories according to the among-module (Pi) and within-module (Zi) connectivity].

The Z–P plot in Figures 6A,B shows the distinct topological roles of different nodes in networks to obtain new insights into the key genus of the three systems (Wang X. et al., 2019). The majority of nodes in bacterial and fungal communities were peripheral. The results showed that 23 bacterial and 9 fungal nodes (except for unassigned OTUs) sink into “connectors” and iron scraps and its types significantly affected the amount of nodes. The details of these connectors are summarized in Supplementary Table 3. Eight connectors detected in the control network (control group) of the bacterial community were OTUs 51,117, 28, 7, 78, 62, 394, and 4. OTUs 51 and 28 were related to *WCHB1*-32. The same number of connectors was detected in the R_Rusty_ system (OTUs 32, 40, 37, 62, 66, 82, 67, and 35). OTU 35 was related to *Aeromonas*, which is identified as a DIRB. Few connectors were detected in the R_Clean_ system (OTUs 4, 95, 26, 67, 69, and 13). OTU 4 belongs to the genus *Saccharimonadales.* These genera showed a positive response to HA performance, thereby demonstrating that these keystone species play important roles in the HA process. Connectors 7, 2, and 0 for the fungal community were detected in the three systems. Members from Ascomycota and Basidiomycota were identified as keystone fungal taxa. The shared connector (OTU 1) was derived from *Apiotrichum*, which was approved to show a relative response to HA performance, especially for acetate generation. In conclusion, only a few connectors were shared between the control group and HA systems with additional iron scraps. This finding suggested that iron scraps significantly alter key microbial populations and the network structure.

### Fate of different iron scraps in the hydrolysis–acidification process

**FIGURE 7:**
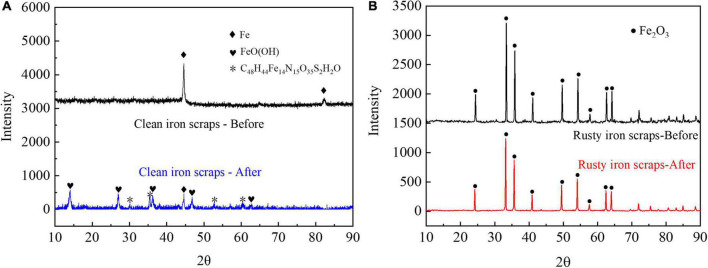
XRD images: iron scraps in **(A)** R_Clean_ and **(B)** R_Rusty_ systems before and after usage.

The solution TFe (in the form of Fe^2+^ because of the anaerobic environment) concentration in HA systems were determined to investigate the fate and role of different iron scraps in the HA process further (Supplementary Figure 2). XRD analysis was applied to analyze chemical compositions of the iron scrap surface (Figures 7A,B). The TFe content in the solution increased to 114.03 mg/L on the 25th day and then decreased to a stable value of 77.64 mg/L in the R_Clean_ system. TFe was released through micro-electrolysis, and passivation of the iron scrap surface was responsible for the decrease of released iron. This finding is consistent with the XRD analysis results. Figure 7A presents the XRD results of iron scraps in the R_Clean_ system. Fe was the main form on the surface of clean iron scraps before usage, while FeOOH, Fe, and C_48_H_44_Fe_14_N_15_O_35_S_2_H_2_O were dominant on the surface of clean iron scraps after usage. Guo et al. (2020) and Tian et al. (2021) reported the existence of FeOOH when iron foam and Fe(II) coupled in the biological systems. These results indicated that sludge can facilitate the oxidation of iron scraps. Meanwhile, C_48_H_44_Fe_14_N_15_O_35_S_2_⋅H_2_O (PDF: 46–1543) was identified and likely a mixture of PNs, DNA, and other biological molecules on the surface of iron scraps.

The TFe content in the solution of the R_Rusty_ system increased continuously likely due to the dissimilatory iron reduction caused by DIRB rather than a pH of 5–8. The XRD results in Figure 7B demonstrated that only diffraction peaks of Fe_2_O_3_ are observed in rusty iron scraps before and after usage. DIRB can successfully reduce insoluble Fe_2_O_3_ through the EET process accompanied by the consumption of Fe_2_O_3_ and release of Fe^2+^ (Bose et al., 2009; Zhou et al., 2017; Shi et al., 2019). Compared with Fe_2_O_3_ powder dosing, rusty iron scraps installed in the middle of reactors in the R_Rusty_ system can supplement the Fe_2_O_3_ layer in a timely manner by exposing to air when the effluent is replaced to avoid the consumption of Fe_2_O_3_. The existence of Fe_2_O_3_ on the iron scrap surface after usage and the TFe concentration can also verify the theory above. Moreover, rusty iron scraps with relatively lower cost (∼$0.25/Kg) and larger volume were more economical and easily recycled than Fe_2_O_3_ (Ou et al., 2016). This finding demonstrated that rusty iron scraps can be preferentially selected for large-scale application.

## Mechanisms of enhancing the hydrolysis–acidification process using different iron scraps

**FIGURE 8:**
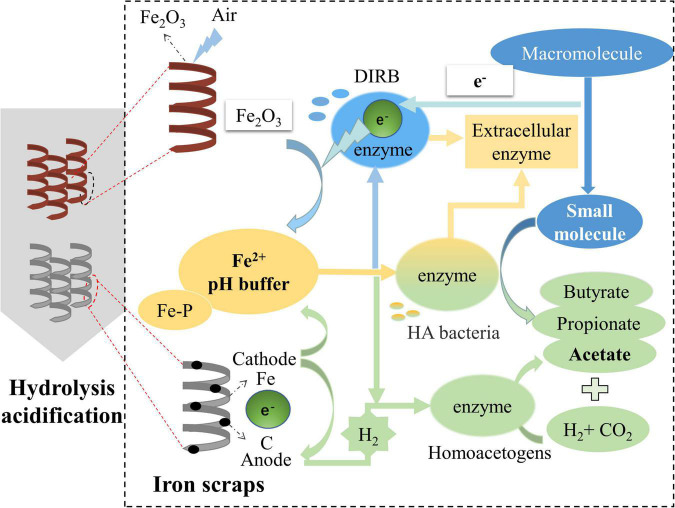
Mechanism of enhancing the HA process using different iron scraps.

The results of this study showed that the HA system is enhanced when iron scraps are added given the higher HE, AE, VFAs production and COD removal efficiency as well as stable pH. HA enhancing mechanisms can be summarized as the enhancement of the system stability and organic transformation ability (Figure 8). Internal reasons for the system stability enhancement were the improvement of EPS generation due to the iron stimulation and the complexity of the microbial network structure.

As for the organic transformation, On the one hand, the generated Fe^2+^ can penetrate cells and promote the synthesis of dehydrogenase which participated in the HA process (Zhu et al., 2014). The mechanisms of Fe^2+^ release was very different in the two iron scrap addition systems. The microbial iron reduction process caused by the DIRB abundance of 6.42% can contribute to reducing the surface-layer Fe_2_O_3_, to generate of Fe^2+^ in the R_Rusty_ system. Whereas iron–carbon micro-electrolysis likely contributed to the generation of Fe^2+^ in the R_Clean_ system. On the other hand, key functional microorganisms were enriched. The relative abundance of bacterial and fungal microorganisms with a positive response to the HA performance increased significantly in HA systems with added iron scraps (sections “Bacterial community and Fungal community”). The relative abundance of the bacterial genus with a positive response to the HA system was 4.42, 24.13, and 21.67%, and the tendency of the fungal genus was the same as that of bacteria. Notably, except for the difference between control and iron scraps added HA systems, the reasons that R_Rusty_ was slightly different from R_Clean_ system was also due to other special enriched genera. The DIRB abundance of 6.42% and relative abundance of 1.16% of homoacetogens were enriched in R_Rusty_ and R_Clean_ systems, respectively. *Aeromonas*, a type of DIRB that can participate in reducing the surface-layer Fe_2_O_3_ and decomposing the macromolecule to small organics, was also identified as a key stone species in section “Iron scrap addition altered microbial co-occurrence networks.” Although *Treponema*, a homoacetogen that can produce acetate by utilizing H_2_ and CO_2_ showed a positive response to the HA performance, it was not identified as a key stone species and the relative abundance of it was too low. Thus, R_Rusty_ was a more effective pretreatment system than R_Clean_ given the macroscopic HA performance and the microscopic microorganism community structure.

## Conclusion

This work demonstrated that the addition of both rusty and clean iron scraps can enhance the HA performance when considering HE, AE, VFAs ratio, and system stability. The internal enhanced mechanisms can be summarized from the aspects of sludge characteristics and microbial community. Rusty and clean iron scraps enriched the microbial genera with their positive response to HA performance, among which the relative abundance of bacterial genera was promoted by 19.71 and 17.25%, respectively. The complexity of the interaction network was increased to enhance the system stability because the total edges of microbial networks were raised. As for the difference between two iron scraps addition HA systems, others functional microorganisms (DIRB and homoacetogens) were also be regarded as main reasons. This study provided new and important insights into the responses of microbial community structures and their MENs to iron scraps in HA systems.

## Data availability statement

The original contributions presented in this study are included in the article/Supplementary material, further inquiries can be directed to the corresponding author/s.

## Author contributions

YW: data analysis and original draft writing, writing—review and editing, and conceptualization. HW: writing—review and editing and conceptualization. HJ: investigation and conceptualization. HC: reviewing and editing and conceptualization. All authors contributed to the article and approved the submitted version.

## Supplementary material

Supplementary Figure 1 SEM images and EDS spectra of sludge samples in **(A,B)** control, **(C,D)** R_Rusty_, and **(E,F)** R_Clean_ systems.

Supplementary Figure 2 HA performance. **(A)** TP concentration; **(B)** TFe concentration in control, R_Rusty_, and R_Clean_ systems.
