# Supplementary material for: Performance and mechanisms of enhanced hydrolysis acidification by adding different iron scraps: Microbial characteristics and fate of iron scraps
Source: Front Microbiol. 2022 Aug 24;13:980396. doi: 10.3389/fmicb.2022.980396 (PMC9449731; doi:10.3389/fmicb.2022.980396)
Supplement: Supplementary file 1 [file Table_1.DOCX]

**Table S1 The composite of trace elements of artificial wastewater**

| Trace elements | Concentration（mg/L） |
| --- | --- |
| CaCl_2_·2H_2_O | 8 |
| MgSO_4_·7H_2_O | 10 |
| FeCl_3_·6H_2_O | 2 |
| CoCl_2_·6H_2_O | 2 |
| ZnCl_2_ | 0.05 |
| CuCl_2_·2H_2_O | 0.05 |
| AlCl_3_·6H_2_O | 0.09 |
| MnCl_2_·4H_2_O | 0.05 |

**Table S2 EPS of sludge in three systems at the end of experiments**

|  | **Control system** | **R_Rusty_ system** | **R_Clean_ system** |
| --- | --- | --- | --- |
| PS (mg/gVSS) | 32.23 | 31.85 | 31.93 |
| PN (mg/gVSS) | 14.09 | 17.39 | 17.38 |
| EPS (mg/gVSS) | 46.32 | 49.24 | 49.31 |

**Table S3. The keystone taxa identified as connectors in bacterial and fungal networks**

|  | OTU | Zi | Pi | Phylum | Genus | Systems |
| --- | --- | --- | --- | --- | --- | --- |
| Bacteria | OTU51 | 1.18 | 0.66 | *Bacteroidetes* | *WCHB1-32* | Control group |
|  | OTU117 | 1.18 | 0.66 | *Bacteroidetes* | *Bacteroidetes_vadinHA17* |  |
|  | OTU28 | 1.18 | 0.66 | *Bacteroidetes* | *WCHB1-32* |  |
|  | OTU7 | 1.18 | 0.66 | *Bacteroidetes* | *Macellibacteroides* |  |
|  | OTU78 | 1.18 | 0.66 | *Proteobacteria* | *Pseudomonas* |  |
|  | OTU62 | 1.18 | 0.66 | *Spirochaetes* | *MVP-15* |  |
|  | OTU4 | 1.18 | 0.66 | *Patescibacteria* | *Saccharimonadales* |  |
|  | OTU394 | 1.18 | 0.66 | *Spirochaetes* | *uncultured* |  |
|  |  |  |  |  |  | R_Rusty_ system |
|  | OTU32 | 1.23 | 0.65 | *Bacteroidetes* | *Lentimicrobiaceae* |  |
|  | OTU40 | 1.23 | 0.65 | *unclassified* | *unclassified_Bacteria* |  |
|  | OTU37 | 0.92 | 0.65 | *Bacteroidetes* | *Bacteroidetes_vadinHA17* |  |
|  | OTU62 | 0.92 | 0.65 | *Spirochaetes* | *MVP-15* |  |
|  | OTU66 | 1.23 | 0.65 | *Bacteroidetes* | *unclassified_Bacteroidales* |  |
|  | OTU82 | 1.23 | 0.65 | *Bacteroidetes* | *Blvii28_wastewater-sludge_group* |  |
|  | OTU67 | 0.00 | 0.66 | *Bacteroidetes* | *Lentimicrobium* |  |
|  | OTU35 | 0.00 | 0.66 | *Proteobacteria* | *Aeromonas* |  |
|  |  |  |  |  |  |  |
|  | OTU4 | 1.22 | 0.65 | *Patescibacteria* | *Saccharimonadales* | R_Clean_ system |
|  | OTU95 | 1.22 | 0.65 | *Bacteroidetes* | *Lentimicrobiaceae* |  |
|  | OTU26 | 1.22 | 0.65 | *Elusimicrobia* | *Candidatus_Endomicrobium* |  |
|  | OTU67 | 0.64 | 0.64 | *Bacteroidetes* | *Lentimicrobium* |  |
|  | OTU69 | -0.24 | 0.65 | *Proteobacteria* | *Desulfobulbus* |  |
|  | OTU13 | -0.24 | 0.65 | *WPS-2* | *WPS-2* |  |
|  | OTU87 | 1.29 | 0.75 | *Ascomycota* | *unclassified* |  |
| Fungi | OTU150 | 1.29 | 0.75 | *Ascomycota* | *Fusarium* | Control group |
|  | OTU1 | 0.00 | 0.73 | *Basidiomycota* | *Apiotrichum* |  |
|  | OTU3 | 1.00 | 0.73 | *Ascomycota* | *unclassified* |  |
|  | OTU8 | 1.00 | 0.73 | *Ascomycota* | *unclassified* |  |
|  | OTU21 | 0.00 | 0.71 | *Ascomycota* | *unclassified* |  |
|  | OTU125 | 0.00 | 0.71 | *Ascomycota* | *Arthrobotrys* |  |
|  |  |  |  |  |  |  |
|  | OTU1 | 0.28 | 0.65 | *Basidiomycota* | *Apiotrichum* | R_Rusty_ system |
|  | OTU150 | 0.28 | 0.65 | *Ascomycota* | *Fusarium* |  |
|  |  |  |  |  |  |  |
