# Supplementary figures and images for: Performance and mechanisms of enhanced hydrolysis acidification by adding different iron scraps: Microbial characteristics and fate of iron scraps
Source: Front Microbiol. 2022 Aug 24;13:980396. doi: 10.3389/fmicb.2022.980396 (PMC9449731; doi:10.3389/fmicb.2022.980396)

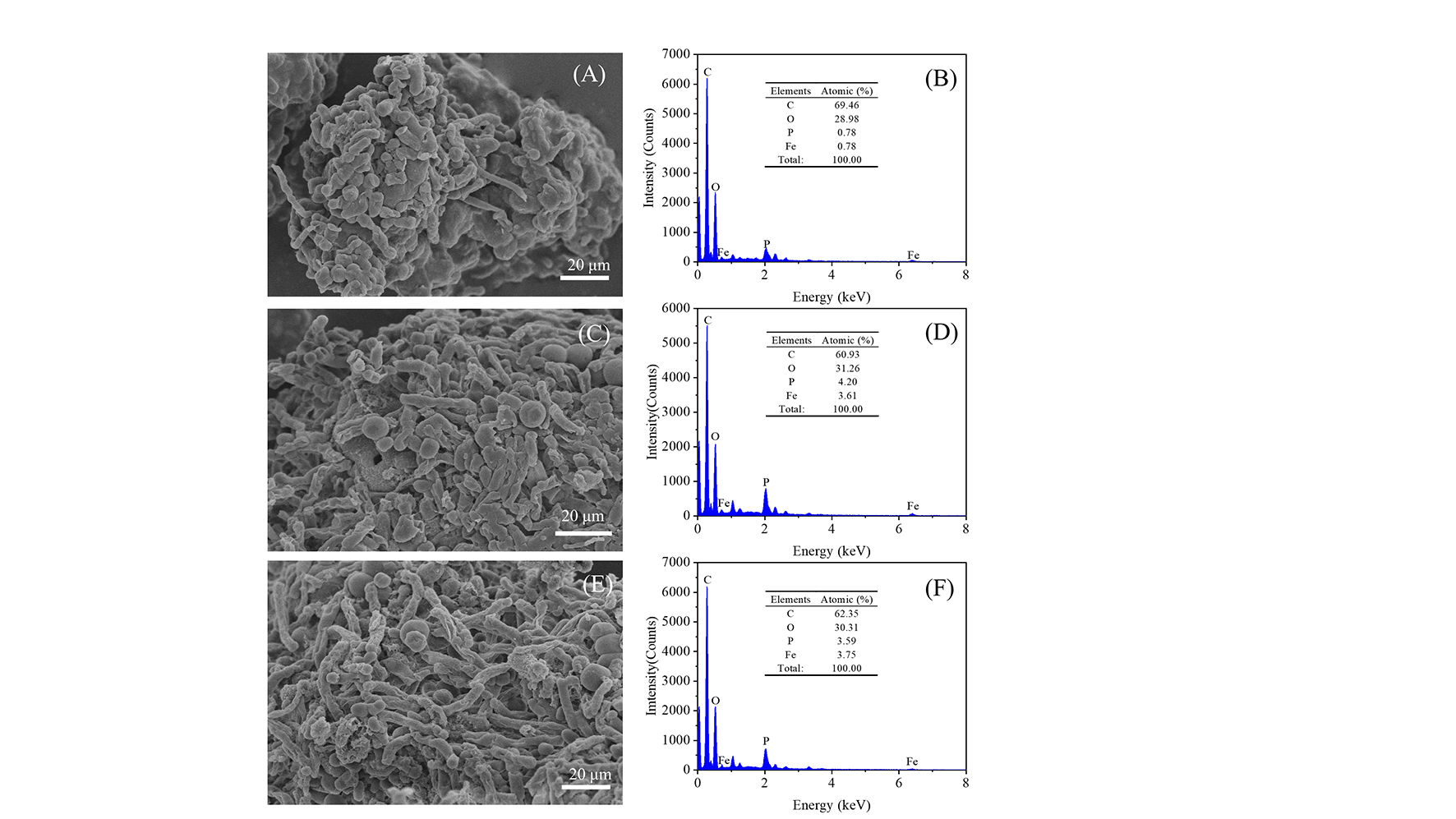

Supplement: Supplementary Figure 1 — SEM images and EDS spectra of sludge samples in (A,B) control, (C,D) RRusty, and (E,F) RClean systems. [file Image_1.TIF]

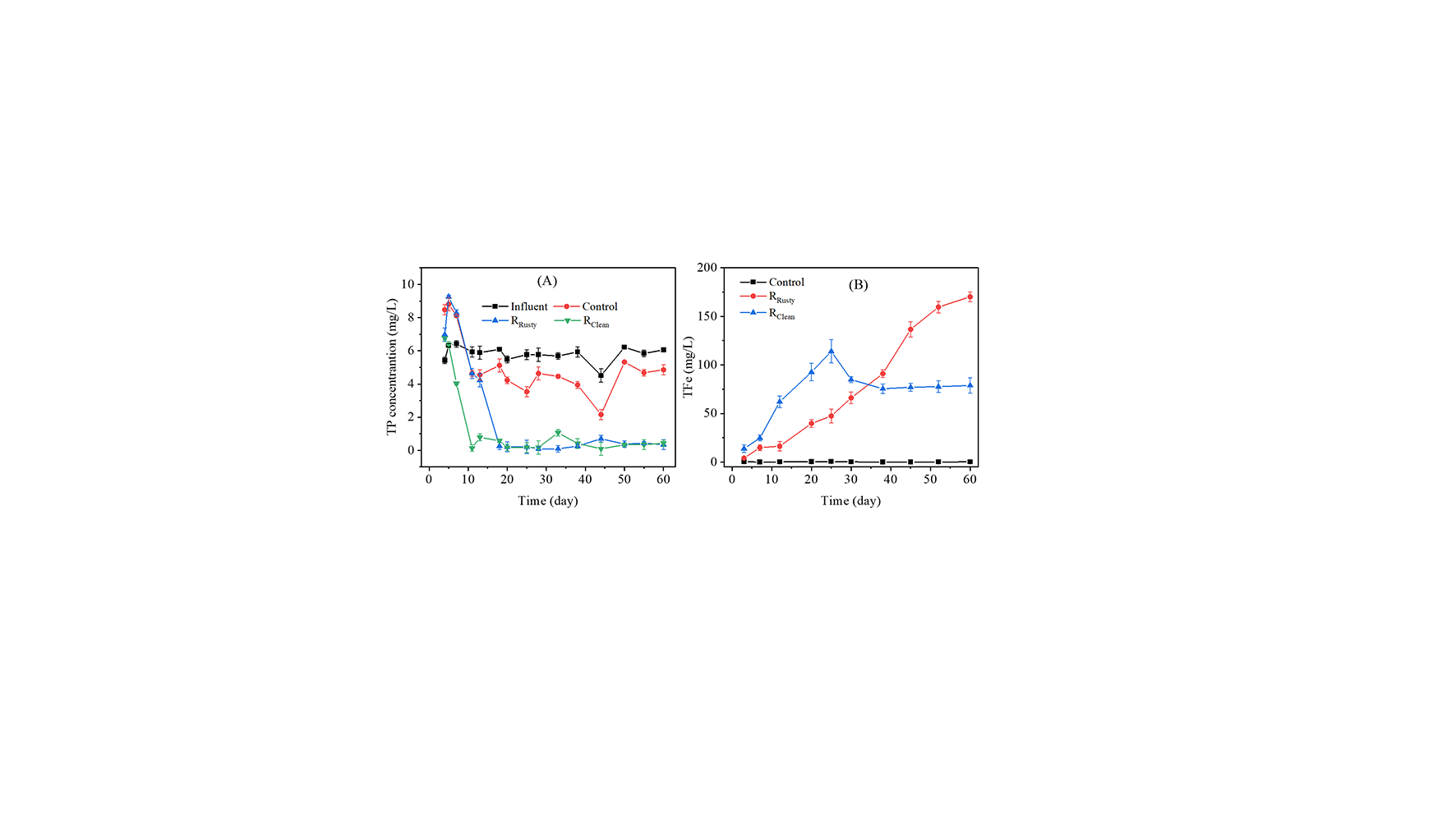

Supplement: Supplementary Figure 2 — HA performance. (A) TP concentration; (B) TFe concentration in control, RRusty, and RClean systems. [file Image_2.TIF]
